# Graph-Based Approaches Significantly Improve the Recovery of Antibiotic Resistance Genes From Complex Metagenomic Datasets

**DOI:** 10.3389/fmicb.2021.714836

**Published:** 2021-10-06

**Authors:** Daria Shafranskaya, Alexander Chori, Anton Korobeynikov

**Affiliations:** ^1^Scientific Center for Information Technologies and Artificial Intelligence, Sirius University of Science and Technology, Sochi, Russia; ^2^Center for Algorithmic Biotechnology, Saint Petersburg State University, Saint Petersburg, Russia; ^3^ITMO University, Saint Petersburg, Russia

**Keywords:** antibiotic resistance, assembly graphs, metagenome, profile hidden Markov model, computational pipeline

## Abstract

The lack of control over the usage of antibiotics leads to propagation of the microbial strains that are resistant to many antimicrobial substances. This situation is an emerging threat to public health and therefore the development of approaches to infer the presence of resistant strains is a topic of high importance. The resistome construction of an isolate microbial species could be considered a solved task with many state-of-the-art tools available. However, when it comes to the analysis of the resistome of a microbial community (metagenome), then there exist many challenges that influence the accuracy and precision of the predictions. For example, the prediction sensitivity of the existing tools suffer from the fragmented metagenomic assemblies due to interspecies repeats: usually it is impossible to recover conservative parts of antibiotic resistance genes that belong to different species that occur due to e.g., horizontal gene transfer or residing on a plasmid. The recent advances in development of new graph-based methods open a way to recover gene sequences of interest directly from the assembly graph without relying on cumbersome and incomplete metagenomic assembly. We present GraphAMR—a novel computational pipeline for recovery and identification of antibiotic resistance genes from fragmented metagenomic assemblies. The pipeline involves the alignment of profile hidden Markov models of target genes directly to the assembly graph of a metagenome with further dereplication and annotation of the results using state-of-the art tools. We show significant improvement of the quality of the results obtained (both in terms of accuracy and completeness) as compared to the analysis of an output of ordinary metagenomic assembly as well as different read mapping approaches. The pipeline is freely available from https://github.com/ablab/graphamr.

## Introduction

Antimicrobial resistance (AMR) is a global health crisis resulting from widespread and uncontrolled use of antibiotics (Brown and Wright, 2016). Therefore, the use of genome sequencing as a surveillance tool for AMR molecular epidemiology is growing, and the development of new computational approaches is an important task (McArthur and Wright, 2015).

Certainly, there are many tools developed recently for AMR prediction and analysis from WGS data (Boolchandani et al., 2019). In general, all these tools could be splitted into two groups: ones that use raw sequencing reads as input, such as SRST2 (Inouye et al., 2014) that use paired-end-aware short read aligner to align reads to reference databases or first splitting reads into k-mers and then aligning them to databases such as KmerResistance (Clausen et al., 2016). Another group of tools that use assembled genome fragments includes Abricate (https://github.com/tseemann/abricate), RGI (Jia et al., 2017), Resfinder (Bortolaia et al., 2020) among the others. ARIBA (Hunt et al., 2017) and RGI (Jia et al., 2017) could utilize both reads and assembled fragments, however, this does not change in general their approach for AMR prediction.

**Figure 1:**
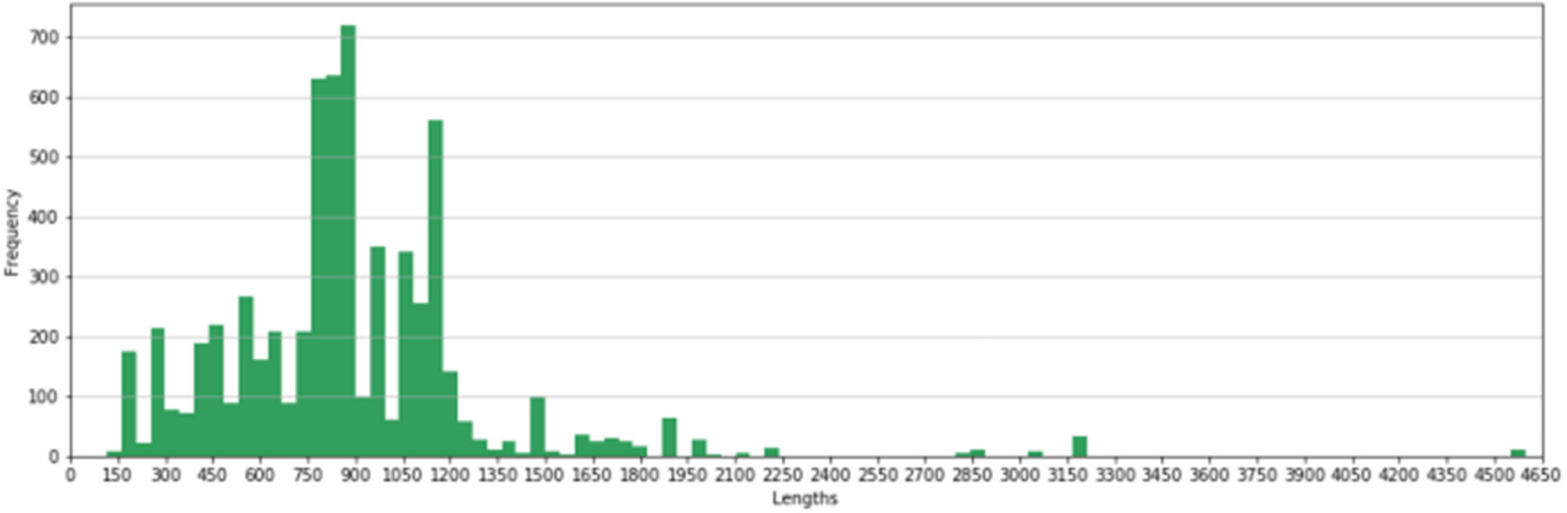
Distribution of AMR gene lengths in the NCBI AMR database.

The natural limitation of any read-based approach is the input read length and therefore the precision of such approach might suffer from the truncated read-gene mappings (depending on the target AMR gene length). Figure 1 shows the distribution of AMR gene lengths in the NCBI AMR database (Feldgarden et al., 2019) with the majority of genes, namely 93%, that are more than 300 base pairs long. Given that typically the reads produced by short reads technologies are within 100–300 bp length, the read-based methods would need to cope with incomplete alignments of reads to AMR databases or additional techniques (e.g., overlapping paired-end reads) would be required in order to correctly cover the genes of interest.

Another approach involves the use of sequences obtained from raw reads after the genome assembly process. Genome assembly may overcome the difficulties connected with the lengths of short reads and allows for reconstruction of fuller gene sequences, however it still has some limitations on its own. Possible issues include possible assembly artifacts, increased computational processing time, etc. Nonetheless, all these issues could certainly be detected, most of them solved in automatic fashion and therefore AMR prediction on top of microbial isolate assembly could be considered a mostly solved problem.

However, the overall situation is much worse when one would need to analyse a resistome from an environmental sample, such as water metagenome, or human-associated sample, e.g., gut metagenome. Such assemblies are often very fragmented due to vastly different species abundance, presence of multiple strains, interspecies repeats that arise from conservative genes or genes that underwent horizontal transfer, etc. (Lapidus and Korobeynikov, 2021). Even more, metagenomic assemblers typically yield a consensus assembly (Nurk et al., 2017) with collapsed strain variations complicating the necessary prediction.

As a result, AMR prediction from metagenomic assembly can show quite low specificity with many important AMR genes unnoticed (Maguire et al., 2020).

**Figure 2:**
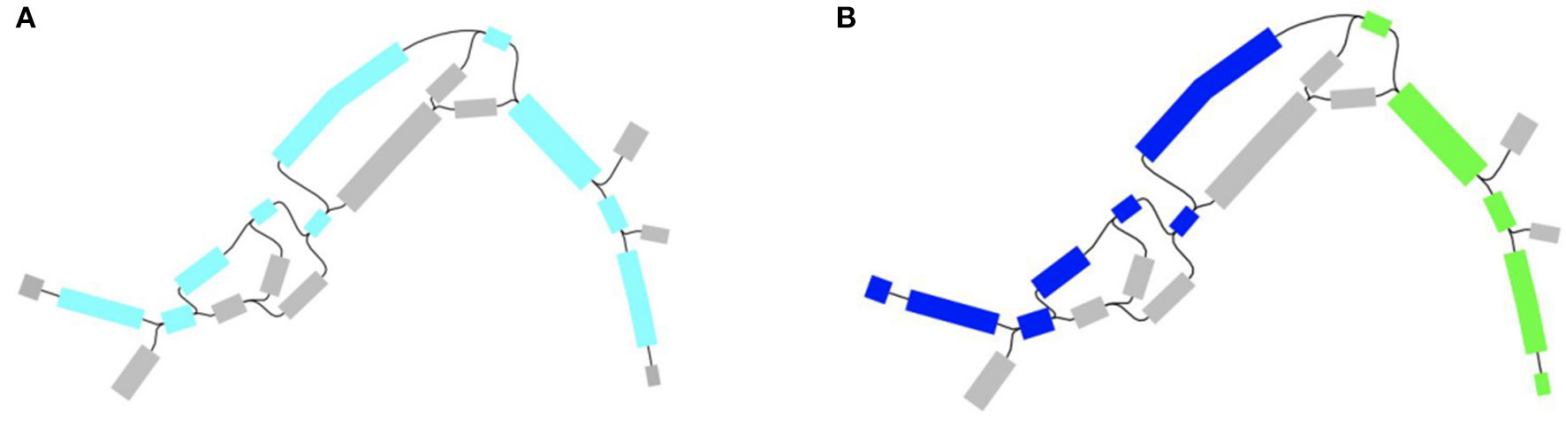
bla_IMP_ sequence in the assembly graph. **(A)** Gene sequence scattered over 10 edges, **(B)** Gene sequence is splitted across two scaffolds.

To support this claim we analyzed wastewater and urban surface metagenomes in Singapore from Ng et al. (2017) that originally used a read-based approach to construct a resistome. First example deals with bla_IMP_ beta-lactamase gene that according to Ng et al. (2017) was absent in the sample. This is not unexpected given the length of bla_IMP_ gene cassette of 741 bp (encoding 246 amino acid polypeptide) (Silva et al., 2002) that certainly could escape from read-based analysis. Furthermore, additional analysis shows that the complete sequence of bla_IMP_ is absent in assembled scaffolds as well, however the bla_IMP_ gene sequence is definitely present in the sample. This phenomenon could be easily explained by examining the assembly graph. Figure 2 shows that the gene sequence of bla_IMP_ is contained in 10 edges of the assembly graph and 2 scaffolds, hindering assembly-based analysis.

**Figure 3:**
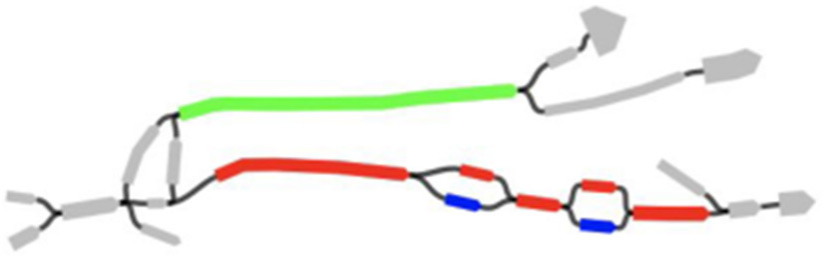
bla_CTX−M_ paths and their neighborhood. Green path corresponds to CTX-M-15 variant; blue and red corresponds to CTX-M-9 and CTX-M-14, respectively.

Sometimes, the gene of interest could be found in contigs, however, when multiple variants are present, not all of them could be easily identified from the contigs alone. Figure 3 shows different variants of the bla_CTX−M_ gene in the assembly graph of the same sample from Ng et al. (2017). We note that CTX-M-15 variant of the gene is residing on the single contig and therefore could be easily identified. However, CTX-M-9 and CTX-M-14 variants differ only by 2 amino acids and therefore assembler is unable to separate them: CTX-M-14 is scattered across 3 contigs that are joined into single scaffold with gaps and CTX-M-9 is completely unassembled as its variation with respect to CTX-M-14 is reported as separate short contigs.

The examples shown above suggest the use of the assembly graph for AMR prediction from complex metagenome sequences since it is the assembly graph rather than set of contigs that represents the “complete” metagenomic assembly result. Even more, metagenomic assemblers provide both so-called strain assembly graph with strain variants preserved and consensus assembly graph with strain variants collapsed (Lapidus and Korobeynikov, 2021), so one could control the tradeoff between specificity and complexity of the task.

**Figure 4:**
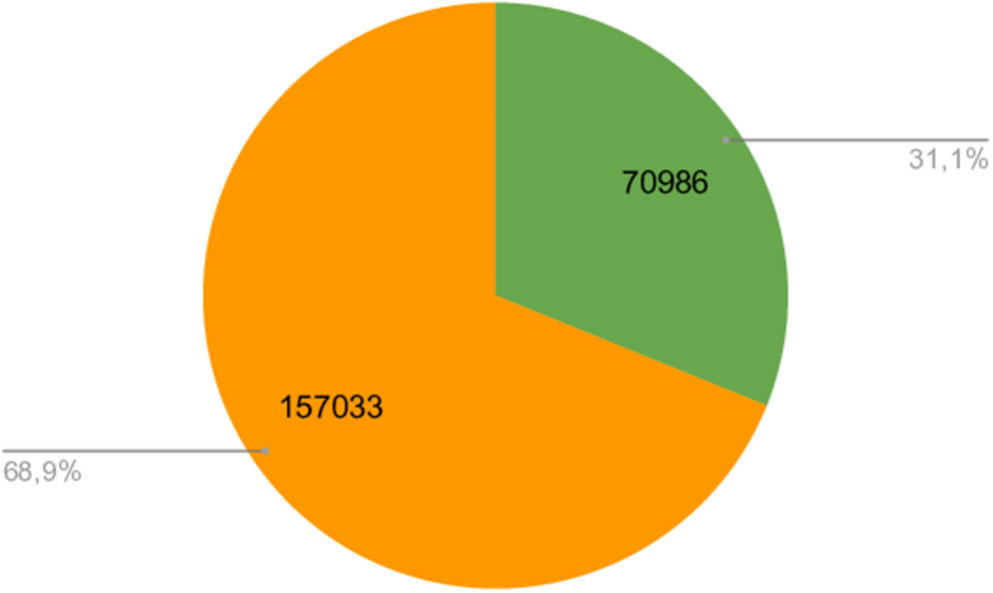
Number of total HMM alignments to graph. The orange section shows the number of HMM hits residing on the scaffolds, and the green section shows the number of HMM hits possibly scattered over multiple scaffolds.

Finally, to show the possible performance gains from assembly graph-based approaches we used PathRacer (Shlemov and Korobeynikov, 2019), a tool that performs profile HMM alignment to assembly graphs, to align NCBI-AMR (Feldgarden et al., 2019) set of AMR profile HMMs to the assembly graphs of samples from Ng et al. (2017) and counted the fraction of HMM hits that are not residing on the single scaffold. Figure 4 shows the results obtained. Overall, more than 30% of all HMM hits are not contained in the single scaffold supporting the idea of using graph-based tools for AMR prediction.

Motivated by the data shown above we are presenting GraphAMR—a novel computational pipeline that utilizes assembly graph of a metagenome for AMR prediction. GraphAMR uses state-of-art tools to align profile HMMs representing AMR gene families, extract the sequences of graph edges that contain HMM hits and uses well-known AMR-prediction tools to further annotate the obtained sequences.

## Pipeline Architecture

GraphAMR is a pipeline specifically designed for recovery and identification of antibiotic resistance genes from fragmented metagenomic assemblies. Briefly, it uses state-of-the-art assembly graph analysis methods to extract putative AMR gene sequences from the graph, dereplicates them and delegates the task of actual prediction to the well-known AMR analysis tools in the field.

The pipeline is implemented using the Nextflow framework (Di Tommaso et al., 2017; Ewels et al., 2020) that enables scalable, reproducible and efficient computational workflow. As a result, the pipeline supports e.g., job submissions on computational clusters and cloud systems, resume, and notification straight out of the box.

**Figure 5:**
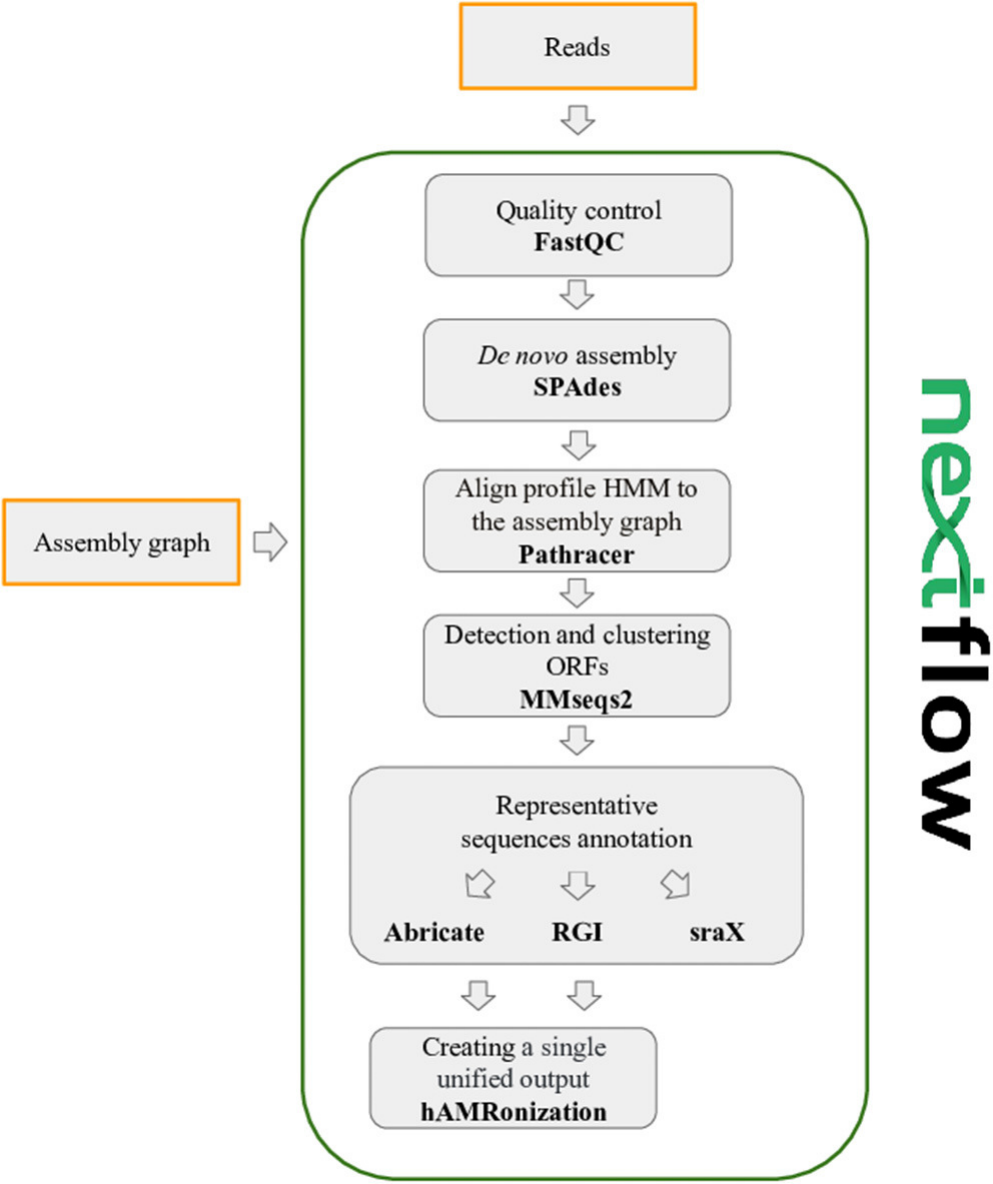
General GraphAMR pipeline scheme.

The pipeline has four steps: (optional) metagenomic *de novo* assembly, alignment of AMR profile HMM to the resulting assembly graph, detection, and clustering of putative AMR ORFs and annotation of representative AMR sequences (Figure 5). The first step (assembly) can be skipped, should the assembly graph in the GFA (https://github.com/GFA-spec/GFA-spec) format be provided as an input. Such assembly graphs are readily produced by genome and metagenome assemblers including SPAdes (Prjibelski et al., 2020), metaSPAdes, and MEGAHIT (Li et al., 2015).

### *De novo* Assembly

If reads are provided as input, the first step will be quality control and metagenomic assembly. Sequences QC is performed via FastQC (https://www.bioinformatics.babraham.ac.uk/projects/fastqc/). The resulting HTML report shows summary graphs with main characteristics for quality assessment. Metagenome assembly is done via metaSPAdes (Nurk et al., 2017) and the resulting assembly graph is used for further analysis.

### Profile HMM or AA Sequence Alignment to Assembly Graph

This is the key step of the pipeline as putative AMR gene sequences are extracted directly from the assembly graph. For this the pipeline utilizes Pathracer (Shlemov and Korobeynikov, 2019), a state-of-the-art tool for alignment of HMMs and AA sequences to assembly graph. By default, the NCBI AMR (Feldgarden et al., 2019) profile HMMs are used, but they could be replaced by the custom HMMs or gene AA sequences if necessary. Pathracer produces the set of most probable paths traversed by a HMM through the whole assembly graph (by default, up to top 100 by score non-redundant paths, e.g., those that are not proper suffixes or prefixes of each other, are reported). This effectively solves the problem of fragmented metagenome assemblies as all possible HMM paths (spanned over multiple contigs) are reported including possible variations due to multiple strains present, interspecies repeats, etc.

The major caveat here is that HMM alignment does not yield the complete gene sequence, since, for example, HMM could be built from the truncated seed alignment, or the alignment itself could be clipped on the ends. To solve this problem, instead of alignment itself, we extract the sequence of graph edges that contain the alignment of interest, effectively extending the alignment until the edge boundaries.

The output of this stage is the set of unique edge sequences of the assembly graph containing the alignments of profile HMMs of AMR genes.

In addition to HMMs, the pipeline also allows alignment of amino-acid sequences to the graph enabling the use of such AMR databases as CARD (Jia et al., 2017) or ResFinder (Bortolaia et al., 2020) directly. To enable the use of such databases, PathRacer internally builds a “proxy” HMM, so that the alignment of this HMM would be equivalent to the alignment of the original sequence using BLOSUM62 scoring matrix.

### Dereplication

The output of the previous step might be redundant due to strain variations, but more because different edge sequences through the assembly graph might yield the same set of genes in the case when alignment ends in the node of the graph (recall that assembly graph is a de Bruijn graph, where subsequent edges overlap by a k-mer) or if there are multiple paths due to synonymous substitutions. To dereplicate the results, the complete ORFs are extracted and further clustered at 90% AA IDY using MMseqs2 (Steinegger and Söding, 2017). The output of this step is the set of representative sequences of the resulting clusters. The dereplication and clustering could be skipped via setting the IDY clustering threshold as 100%.

### Annotation

There is no need to design a completely new AMR prediction approach given that the major challenges of obtaining putative AMR sequences from fragmented metagenome assemblies are solved via the proper utilization of the assembly graph. Therefore, this step delegates the task of final AMR prediction, annotation, and result generation to state of the art tools that are well-known and respected by the bioinformatics community. The pipeline passes the output of the dereplication stage to abricate (https://github.com/tseemann/abricate), sraX (Panunzi, 2020), and rgi (Jia et al., 2017). The results are further combined and summarized by hAMRonize tools (https://github.com/pha4ge/hAMRonization).

## Results

### Usage

The pipeline is implemented in Nextflow and therefore requires Nextflow to be installed in order to be used. For the full reproducibility, the use of Nextflow-supported package manager such as Conda is advised. GraphAMR will automatically pull the necessary versions of the tools used in the pipeline when using one of the supported container engines. The typical steps to run the pipeline for the first time are as follows:

1. Install nextflow (https://nf-co.re/usage/installation)
2. Install any Nextflow-supported container engines, such as conda (https://conda.io/miniconda.html)
3. Download the pipeline and test it on a minimal dataset with a single command: nextflow run ablab/graphamr -profile test, conda
4. Start running your own analysis:
  - Typical command for analysis starting from reads (NCBI AMR database is used by default):
nextflow run ablab/graphamr -profile conda --reads ‘^*^_R{1,2}.fastq.gz’
  - Typical command for analysis starting from assembly graph (NCBI AMR database is used by default):
nextflow run ablab/graphamr -profile conda --graph ‘assembly_graph_with_scaffolds.gfa’
  - Typical command for analysis starting from assembly graph with one of pre-defined AMR databases:
nextflow run ablab/graphamr -profile conda --graph ‘assembly_graph_with_scaffolds.gfa’ --db [‘ncbi_AMR_HMM’, ‘card_AA’]

More examples, description of other command line options and produced results are available from the “Usage/Results” section of documentation in GraphAMR github repository.

### Example Results

To demonstrate the performance of graph-based approach for AMR discovery we benchmarked GraphAMR pipeline on two different environmental datasets using two different databases: NCBI AMR HMMs and amino acid sequences from CARD.

URBAN is a collection of urban wastewater datasets from Ng et al. (2017). Raw sequence reads were downloaded from the NCBI short read archive (SRA) under accession numbers SRR5997540–SRR5997552 and analyzed using the pipeline. For the sake of simplicity only AMR predictions by Abricate are shown. Table 1 contains the predicted AMR gene counts predicted from metagenomic assembly scaffolds, unclustered HMM paths and HMM paths dereplicated, and clustered at different IDY's %. The results of the pipeline using amino acids are presented in Table 2.

**Table 1:** Abricate predicted AMR gene sequence counts in the URBAN dataset.

**Sample ID**	**40**	**41**	**42**	**43**	**44**	**45**	**46**	**47**	**48**	**49**	**50**	**51**	**52**
Contigs	92	93	2	0	4	9	57	0	91	3	11	78	66
HMM Paths	169	163	2	0	4	8	100	0	131	3	22	122	142
Clustered ORFs (90%)	103	98	2	0	4	8	60	0	96	3	11	91	80
Clustered ORFs (95%)	105	105	2	0	4	8	61	0	100	3	11	92	81
Clustered ORFs (100%)	135	126	2	0	4	8	75	0	112	3	14	107	116

*Compared are assembled contigs, unclustered HMM paths, and clustered ORFs at different levels of IDY's. Columns are named by the last two digits of SRA accession number*.

**Table 2:** Abricate predicted unique AMR gene sequence counts in the URBAN dataset using amino-acid sequences from CARD v3.1.2 or HMMs from NCBI AMR to align to a graph.

**Sample ID**	**40**	**41**	**42**	**43**	**44**	**45**	**46**	**47**	**48**	**49**	**50**	**51**	**52**
AA	96	89	2	0	4	8	59	0	89	3	11	84	74
HMM	94	89	2	0	4	8	59	0	89	3	10	82	74

*Columns are named by the last two digits of SRA accession number*.

The resulting AMR presence heatmap as produced by RGI is available as Supplementary Figure 1. The running time, physical memory usage and CPU usage and graph size information presented in the Supplementary Figure 1 and Table 1, respectively.

We note that HMM paths represent unique path sequences over the assembly graph and might be redundant: two different paths in the graph may yield the same amino acid gene sequence, for example, due to synonymous mutations or if the alignment ends in the node of the graph since edges have overlapping k-mers. This explains the higher number of predicted AMR gene sequences obtained from bare HMM paths as compared to dereplicated or clustered ORFs.

The sample SRR5997545 looks like an outlier in Table 1, as the number of predicted AMR genes out of contigs is higher than from the assembly graph. The difference is caused by the short hit that resides on the isolated edge of the assembly graph. The hit itself covers only 73% of the HMM. By default Pathracer uses the strict threshold and does not report hits that are shorter than 90% of HMM length (we expect fuller HMM matches from the assembly graph as compared to contig sequences). To allow inclusion of such sequences should they be necessary we added a special flag to the pipeline that allows a user to choose the desired HMM coverage threshold.

To further compare the assembly graph-based approach with the read-based one we run SRST2 on the same collection of datasets. Table 3 contains the predicted unique AMR gene counts from raw reads as detected by SRST2 and clustered HMM paths from GraphAMR. SRST2 uses a custom AMR database that was derived from CARD v3.0.8. To ensure fair comparison we run GraphAMR pipeline and Abricate using the database that was used by SRST2.

**Table 3:** Predicted unique AMR gene sequence counts in raw reads of URBAN as detected by SRST2 vs. GraphAMR predictions from the assembly graph.

**Sample ID**	**40**	**41**	**42**	**43**	**44**	**45**	**46**	**47**	**48**	**49**	**50**	**51**	**52**
SRST2	59	55	6	0	2	6	36	0	59	2	8	54	44
GraphAMR	90	83	1	0	3	7	52	0	82	3	10	79	68

*AMR annotation was done via Abricate. All tools used the CARD_v3.0.8_SRST2 database. Columns are named by the last two digits of SRA accession number*.

Table 3 clearly shows the advantage of the graph-based approach since more AMR gene sequences were predicted in almost all samples as compared to the read-based approach. Still, there is one notable outlier: in SRR5997542 sample SRST2 predicted 5 more AMR genes. Further detailed analysis revealed that these hits are likely spurious: the sequences themselves are fragmented on the assembled graph and the graph edges are isolated (see Supplementary Figure 2).

SOIL is groundwater metagenome sample SRR8931193 from Smith et al. (2019). Abricate predicted 12 AMR genes from clustered HMM paths and 13 from assembled scaffolds. Two gene sequences [vanR-O and ant(6)-Ib] genes were found only on scaffolds and tet(X) was detected by GraphAMR only. Assembly graph analysis revealed that ant(6)-Ib gene sequence is split into two parts located on two isolated edges. vanR-O hit covered only 30% of the corresponding sequence and is likely spurious.

## Discussion

As Tables 1–3 and Figure 4 show, the results of AMR gene prediction even on moderately-complex metagenomes could be significantly affected by fragmented assemblies. The use of assembly graph-based approaches is far superior in terms of recovery of fuller AMR gene sequences even from fragmented metagenomes. Not only could it result in more putative AMR sequences detected, but as comparison with read-based approaches shows, the results are more reliable. Graph-based approach allows to filter out the spurious alignments using both hit length (the fraction of the gene sequence length covered by a hit) and graph topology (short hits located on isolated edges are likely spurious) that results in AMR gene sequences that are both longer (hit could span multiple edges and interspecies repeats) and trustworthy (located on the edges of the graph that are connected to the rest of the assembly).

Another important task that could be solved using the assembly-graph based approach is AMR host association: sometimes it is not enough simply to detect the gene sequences, but also associate them with the particular species. This task is quite complex in case of metagenomic assemblies as a dedicated procedure called “binning” is required. However, typically binners ignore short contigs (shorter than 2–5 kbp) and therefore further detection of AMR gene sequences from MAGs could be quite limited (Maguire et al., 2020). Graph-based approach allows to circumvent this problem as one could trace the detected AMR sequences back to the edges of the assembly graph and then to the corresponding MAGs performing the required species identification. The challenge here certainly is dealing with interspecies repeats and/or plasmids or otherwise transferred genes, however, the assembly graph provides a solid foundation for such downstream analysis.

GraphAMR could be used to improve the present results of AMR prediction of a metagenomic assembly if the assembly graph output was preserved, otherwise the pipeline allows for seamless reassembly and AMR prediction starting from the input sequencing reads.

## Data Availability Statement

Publicly available datasets were analyzed in this study. This data can be found here: NCBI SRA: SRR5997540–SRR5997552, SRR8931193.

## Author Contributions

AK contributed to conception and design of the study. AK, AC, and DS implemented the pipeline. AK and DS wrote sections of the manuscript. All authors contributed to manuscript revision, read, and approved the submitted version.

## Funding

This reported study was funded by RFBR, project number 19-34-51017.

## Conflict of Interest

The authors declare that the research was conducted in the absence of any commercial or financial relationships that could be construed as a potential conflict of interest.

## Publisher's Note

All claims expressed in this article are solely those of the authors and do not necessarily represent those of their affiliated organizations, or those of the publisher, the editors and the reviewers. Any product that may be evaluated in this article, or claim that may be made by its manufacturer, is not guaranteed or endorsed by the publisher.
