# Supplementary material for: Graph-Based Approaches Significantly Improve the Recovery of Antibiotic Resistance Genes From Complex Metagenomic Datasets
Source: Front Microbiol. 2021 Oct 6;12:714836. doi: 10.3389/fmicb.2021.714836 (PMC8528159; doi:10.3389/fmicb.2021.714836)
Supplement: Supplementary file 1 [file Data_Sheet_1.pdf]

	Total vertices	Total nucleotides	Total paths	GFA file size
SRR5997540	3034188	1546182930	1466318	424 Mb
SRR5997541	2688536	1355997296	1285372	372 Mb
SRR5997542	2953400	1340318360	1439680	372 Mb
SRR5997543	2515768	1232593374	1230552	339 Mb
SRR5997544	4444240	1781622996	2151588	504 Mb
SRR5997545	3494092	1471059268	1730428	412 Mb
SRR5997546	4056072	1690748804	1987258	478 Mb
SRR5997547	3744292	1551951858	1835650	438 Mb
SRR5997548	2202340	1114128928	1059954	305 Mb
SRR5997549	3428164	1407665344	1664672	397 Mb
SRR5997550	3550116	1495352614	1719294	420 Mb
SRR5997551	2570028	1274530320	1227478	351 Mb
SRR5997552	2865424	1447288162	1364620	397 Mb

Supplementary Table 1. Number of vertices, edges, nucleotides and graph size in each sample from `URBAN` collection

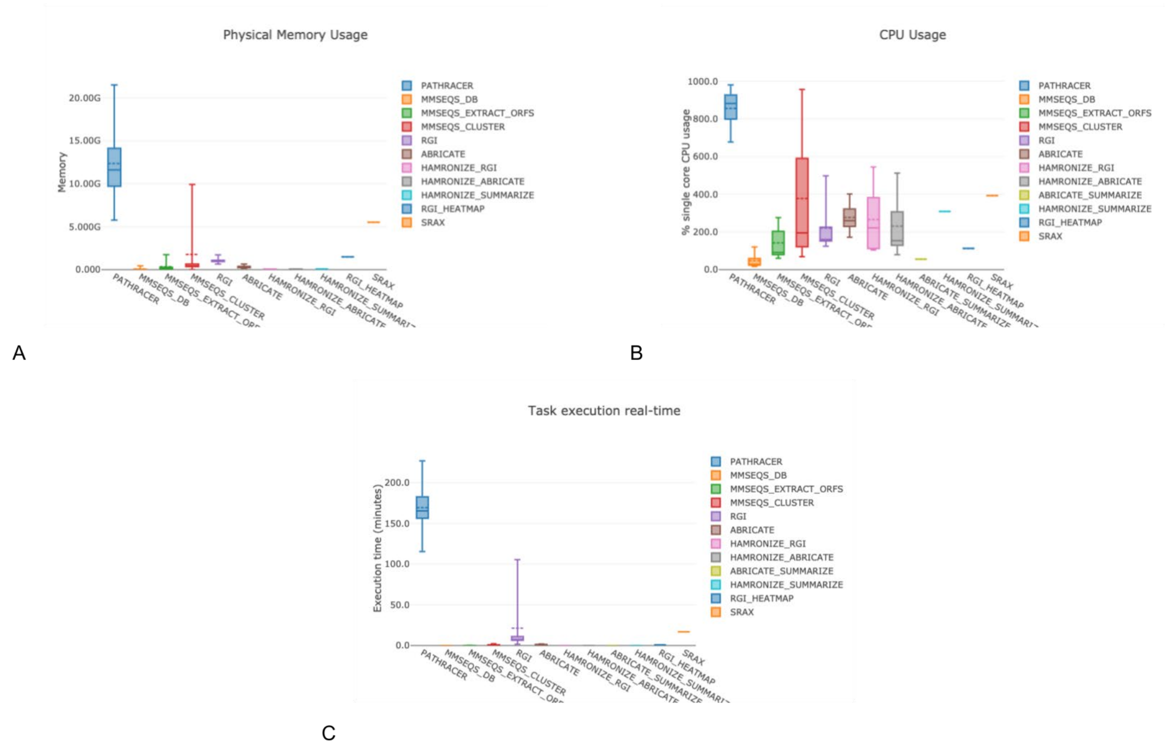

Supplementary Figure 1. Summary information about the performance (memory usage, CPU usage, execution runtime) of the pipeline on `URBAN` collection.

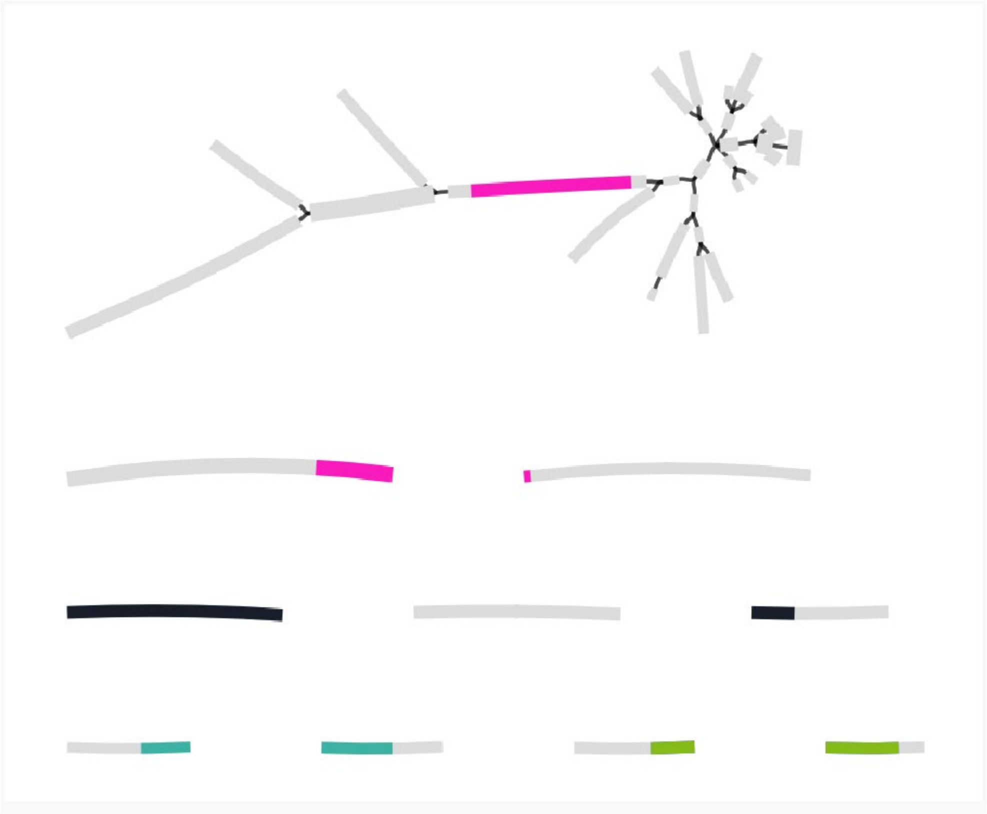

Supplementary Figure 2. Locations of the predicted AMR gene sequences from the reads of the SRR5997542 on the assembly graph. These genes were not detected in clustered HMM paths since the genes are located on different edges that are not related to each other.
